# HDAC1 and SATB1 positively regulate immune responses in chicken macrophages

**DOI:** 10.1016/j.psj.2026.106607

**Published:** 2026-02-10

**Authors:** Bowen Niu, Junda Hu, Zixuan Fan, Zihao Gao, Yuchen Jie, Xinyu Wu, Xingying Chen, Sirui Chen, Li-Wa Shao

**Affiliations:** a State Key Laboratory of Animal Biotech Breeding and Frontier Science Center for Molecular Design Breeding, China Agricultural University, Beijing 100193, China; b Department of Animal Genetics and Breeding, National Engineering Laboratory for Animal Breeding and Key Laboratory of Animal Genetics, Breeding and Reproduction, Ministry of Agriculture and Rural Affairs, College of Animal Science and Technology, China Agricultural University, Beijing 100193, China; c State Key Laboratory of Virology, College of Life Sciences, Wuhan University, Wuhan 430072, China; d Tsinghua Institute of Multidisciplinary Biomedical Research, Tsinghua University, Beijing 102206, China; e National Institute of Biological Sciences, Beijing 102206, China

**Keywords:** Chicken macrophage, Lipopolysaccharide, HDAC1, SATB1, Immune regulation

## Abstract

The avian immune system constitutes the primary barrier against pathogen invasion, and its regulatory efficiency directly determines animal health, productive performance, and food safety. Elucidating the molecular and cellular networks that maintain immune homeostasis in poultry has therefore become a pivotal entry point for improving disease prevention and achieving environmentally sustainable production. Histone deacetylase 1 (HDAC1) and special AT-rich sequence-binding protein 1 (SATB1) are reported modulators of immunity in some species such as mammals, yet their roles in avian species remain undefined. Here, we employed chicken macrophages (HD11) stimulated with lipopolysaccharide (LPS) to establish an in vitro immune-response model. Both *HDAC1* and *SATB1* were markedly activated and up-regulated upon LPS challenge. Using cell transfection and CRISPR/Cas9 genome editing, we generated HD11 cell lines with stable disruption of either *HDAC1* or *SATB1*. In these modified cells, the LPS-induced elevation of key immune effectors—including *IFN-β, IRF7, STAT1, TNF-α*, and *IFIH1* was significantly attenuated. Amino-acid sequence alignment, protein complex prediction, and co-immunoprecipitation further suggest that HDAC1 and SATB1 are evolutionarily conserved and physically interact. Collectively, our study uncovers a novel mechanism by which HDAC1 and SATB1 act synergistically to positively regulate immune responses in chicken macrophages. These findings not only provide new theoretical insights into avian immune regulation but also establishes a molecular foundation for the development of next-generation immune enhancers and breeding strategies that enhance disease resistance.

## Introduction

Intensified poultry production has markedly increased the efficiency of chicken meat and egg output, yet it simultaneously amplifies pathogen exposure within flocks (Gilbert et al., 2017; Van Steenwinkel et al., 2011). High-density housing not only heightens the risk of devastating outbreaks of Newcastle disease, avian influenza, and other highly contagious pathogens (Astill et al., 2018; Guinat et al., 2025), but also triggers a cascade of embryonic heat stress, oxidative damage, and feed-borne immunosuppression that collectively keep birds in a state of chronic immune overload (Emami et al., 2020; Goel, 2021; Oluwagbenga and Fraley, 2023). Consequently, a systematic dissection of the molecular networks governing the avian immune response and their regulatory mechanisms has become pivotal for overcoming current industry bottlenecks: frequent disease episodes, antibiotic overuse, and declining productivity (Ziebe et al., 2025). This endeavor carries both fundamental and forward-looking significance for achieving healthy poultry farming and green, sustainable development (Bist et al., 2024).

The avian immune system is governed by networks of innate-immunity genes (e.g., Toll-like receptors and interferon genes) (Nawab et al., 2019; Wang et al., 2022), adaptive-immunity genes (e.g., T-cell receptor and B-cell receptor genes) (Boodhoo et al., 2022; Kaufman, 2022), and cytokine genes—including interleukins and tumor necrosis factors—that modulate immune-cell functions (Lu et al., 2022; Truong et al., 2017). The chicken macrophage cell line HD11 has been established as a pivotal in vitro model for dissecting avian immune activation and defense mechanisms (Sandholt et al., 2021; Wang et al., 2020). To date, investigators routinely stimulate HD11 cells with lipopolysaccharide (LPS), Toll-like receptor (TLR) agonists, or pathogen infection to construct cellular immune-response paradigms and subsequently assess avian immunocompetence by quantifying the expression of key immune genes such as *TNF-α* and *IFN-β* (Jin et al., 2018; Peroval et al., 2013).

Studies have revealed that histone deacetylase 1 (HDAC1) and special AT-rich sequence-binding protein 1 (SATB1) exert immunoregulatory functions. As a core component of the epigenetic regulatory network, HDAC1 modulates the activation, differentiation, and function of macrophages, T cells, and other immune cells through its deacetylase activity (De Sá Fernandes et al., 2024; Turgeon et al., 2013; Zhu et al., 2024). SATB1 specifically binds to matrix attachment regions (MARs) in the genome and serves as an anchoring point for chromatin organization, bringing distal enhancers, silencers, and other regulatory elements into proximity with target gene promoters to form chromatin loops, thereby efficiently activating or repressing gene transcription (Alvarez et al., 2000; Yasui et al., 2002). SATB1 coordinates the spatiotemporal expression of a wide array of immune-related genes by establishing specific chromatin loop structures in T cells, which in turn regulating T cell activation, differentiation, and function (Qi et al., 2024; Zelenka and Spilianakis, 2020).

In mammals and *Caenorhabditis elegans*, HDAC1 and SATB1 have been shown to form protein complexes and regulate cellular state and immunity, thereby influencing systemic metabolic reprogramming, aging, and even lifespan (Kohwi et al., 2025; Liu et al., 2018; Shao et al., 2020; Yasui et al., 2002). However, whether such an interaction exists in avian species, and whether HDAC1 and SATB1 play similar immunoregulatory roles in avian macrophages, remain poorly understood. Moreover, the evolutionary conservation and functional divergence of this epigenetic regulatory module in birds await systematic investigation.

This study aims to elucidate the regulatory roles of HDAC1 and SATB1 in LPS-triggered immune responses in chickens. First, an LPS-stimulation model was established in HD11 cells to characterize the expression dynamics of HDAC1 and SATB1 during immune activation. CRISPR/Cas9 was then used to generate *HDAC1*- or *SATB1*- disrupted cells, enabling a systematic assessment of how the loss of each gene alters the transcription of key immune effectors after LPS challenge. In parallel, the HDAC1-SATB1 interaction was predicted by in silico analysis and confirmed by co-immunoprecipitation (co-IP). Collectively, these findings advance understanding of HDAC1 and SATB1 function in avian innate immunity and provide both a theoretical framework and prospective targets for modulating immune responses to enhance disease resistance and vaccine efficacy in poultry.

## Materials and methods

### Cell and plasmids

The chicken macrophage cell line HD11 was a generous gift from Prof. Guiping Zhao. The PB-eGFP plasmids was a generous gift from Prof. Sen Wu. The plasmids pX459 and pcDNA3.1 were obtained from Addgene.

### Cell culture and treatment

HD11 cells were cultured in RPMI 1640 (Gibco, #A1049101) medium containing 10% (v/v) fetal bovine serum (Gibco, #10099141) and 1% penicillin-streptomycin (Gibco, #15140122) at 37°C.

For lipopolysaccharide stimulation, cells were seeded in 6-well plates and incubated for 24 h. A 1 mg/mL LPS (Sigma, #L2880) stock solution prepared in PBS (Solarbio, #P1020) was serially diluted in HD11 complete medium to final working concentrations of 0.2, 1, and 5 μg/mL, based on previous studies (Li et al., 2023, 2024; Nam et al., 2025). After 24 h of treatment, cells were imaged with an inverted fluorescence microscope (Olympus, CKX3-SLP) and harvested for gene-expression analysis.

For sodium butyrate treatment, cells were treated with 5 mM sodium butyrate (Sigma, #303410) for 24 h. For combined sodium butyrate and lipopolysaccharide treatment, lipopolysaccharide was added to the culture medium 3 h after the sodium butyrate.

### RNA extraction and RT-qPCR

HD11 cells were harvested and lysed directly into TRIzol reagent (Invitrogen, #15596026). Total RNA was extracted by chloroform phase separation, isopropanol precipitation, and 75% (v/v) ethanol washing (Chomczynski and Sacchi, 1987). RNA concentration was determined with a NanoDrop spectrophotometer. 1 μg of RNA per sample was reverse-transcribed into cDNA using the PrimeScript RT Reagent Kit (Takara, #RR047A). Quantitative PCR was performed with SYBR Premix Ex Taq (Takara, #RR820A). Gene expression was analyzed by the ΔΔCt method with *ACTB* as the endogenous reference. ΔCt for each sample was calculated as Ct_target – Ct_*ACTB*. ΔΔCt was then obtained by subtracting the ΔCt of the untreated control group. Relative fold change was computed as 2^(–ΔΔCt), and values were normalized to the untreated control (set as 1). Primer sequences for real-time PCR are listed in Table S1.

### Expression correlation analyses

Correlations among *HDAC1, SATB1* and the immune genes in the control or LPS-treated HD11 cells were assessed using RT-qPCR measurements. Pearson’s correlation coefficients were calculated and visualized with the plugin Correlation Plot (v1.41) in OriginPro 2025b (OriginLab Corporation, USA).

### Cell transfection

When cells in 6-well plates reached 70–80% confluence, plasmids (e.g., PB-eGFP) were transfected using Starvio transfection reagent (StarVio, #T21002) according to the manufacturer’s instructions. The medium was replaced with HD11 complete medium 24 h post-transfection. Transfection efficiency was assessed 24 h later (e.g., by GFP fluorescence). Transfected cells were then selected with 1 µg mL⁻¹ puromycin (Solarbio, #P8231) for several days to obtain stably transduced populations.

### Cellular gene editing

Target sites for CRISPR/Cas9 were designed using the web tool CHOPCHOP v3 (https://chopchop.cbu.uib.no/). Sites were restricted to exons 1-2, GC content was set to 40–60%, and only off-target sites with ≤2 mismatches in the protospacer were permitted. The top-ranking candidates that met these criteria were selected and verified by sequencing. The target sequences were designed as follows: *HDAC1*, 5′-TGGGTCATCCGGATCCTGT-3′; *SATB1*, 5′-TAACAATGTAAGCGATCCGA-3′. Corresponding single-guide oligos were synthesized, annealed, and ligated into BbsI (New England Biolads, #R3539) -digested, gel-purified pX459 vector. After transformation, the plasmid clones were propagated and verified by Sanger sequencing. Validated plasmids were transfected into cells; 48 h later, puromycin selection was applied, and mixed populations of resistant cells were expanded for follow-up experiments.

### T7EI assay

To quantify gene-editing efficiency, we employed the T7 endonuclease I (T7E1) assay, which exploits the enzyme’s ability to cleave mismatched DNA duplexes (Sentmanat et al., 2018; Vouillot et al., 2015). Genomic DNA was extracted from cells using the TIANamp Genomic DNA Kit (Tiangen, #DP304-03). DNA fragments encompassing the CRISPR/Cas9 target site were PCR-amplified, purified, and subjected to denaturation, re-annealing, and T7E1 digestion according to the manufacturer’s protocol (NEB, #M0302). The resulting products were resolved by agarose-gel electrophoresis to visualize band shifts indicative of indels. Indel efficiency was quantified from the relative band intensities measured with Image J. To determine the exact mutant sequences, the PCR products were cloned into a blunt-end cloning vector (Vazyme, #C601-01), and individual colonies were Sanger-sequenced.

Primer sequences for mutation identification:

*HDAC1*: forward 5′-AAAGCTTTCTGATGGTGCAGA-3′, reverse 5′-ACACACCACATATCTAGGCAAAG-3′;

*SATB1*: forward 5′-AAGGCATCTTCTGCCTTGTTT-3′, reverse 5′-AGCATGAAGCTTACATTTCCCA-3′.

### Nanoliquid chromatography and mass spectrometry analysis

Cells in 10 cm dishes at 90% confluency were rinsed with PBS, scraped, pelleted by centrifugation, and resuspended in a lysis buffer containing 7 M urea and 2 M thiourea for sonication and protein extraction. After quantification by the Bradford assay, 40 μg of protein from each sample was subjected to enzymatic digestion via the FASP method.

DIA acquisition was performed on an Orbitrap Fusion ultrahigh-resolution mass spectrometer (Thermo Scientific, USA) coupled with an ACQUITY UPLC M-Class (Waters, USA). Peptides were spiked with iRT internal standard peptides (Biognosys, Switzerland) and loaded onto an Acclaim PepMap trap column (Thermo Scientific, USA); the peptides were separated using a self-packed analytical column filled with Aqua C18 stationary phase (1.8 μm, 125 Å, Phenomenex, USA). Mobile phase A was water with 0.1% formic acid, and mobile phase B was acetonitrile/water/formic acid (80:19.9:0.1, v/v/v). The peptides were eluted using a 90-min gradient program. All data were acquired in profile mode and analyzed with Spectronaut 18.0 software (Biognosys, Switzerland) against the UniProt_chicken database (2015_06). Trypsin was selected as the proteolytic enzyme with a maximum of two missed cleavages. A decoy database was used, and peptides were filtered at 1% FDR. Proteins were quantified at the MS1 level.

### Functional rescue experiments

*HDAC1* and *SATB1* overexpression plasmids were constructed by inserting the corresponding cDNA sequences into the pcDNA3.1 vector. Synonymous mutations were introduced into the Cas9 target sequences of the gene sequences to prevent CRISPR-mediated cleavage. HD11 cells were either transfected with pX459 plasmid targeting the target gene or co-transfected with pX459 and pcDNA3.1 vector, followed by LPS treatment. Cells were harvested for RNA extraction and RT-qPCR analysis.

### Protein sequence and structure analysis

The amino acid sequences of chicken HDAC1 (NP_989487.1), chicken SATB1 (NP_001186573.1), human HDAC1 (NP_004955.2), and human SATB1 (NP_001124482.1) were retrieved from the NCBI Protein database (https://www.ncbi.nlm.nih.gov/). Sequence alignment was performed with ClustalW (https://www.genome.jp/tools-bin/clustalw) and visualized in ESPript 3.0 (https://espript.ibcp.fr/ESPript/ESPript/) with secondary-structure annotation.

The AlphaFold-predicted three-dimensional structures of chicken HDAC1 (P56517), chicken SATB1 (E1BRH4), human HDAC1 (Q13547), and human SATB1 (Q01826) were obtained from the UniProt database (https://www.uniprot.org/). Protein-protein docking between HDAC1 and SATB1 was performed using the ZDOCK server (https://zdock.wenglab.org/). Additionally, the full-length amino acid sequences of the aforementioned protein pairs from each species were submitted to the AlphaFold Server (https://alphafoldserver.com) for complex structure prediction. All structural models were visualized and analyzed using PyMOL v3.1.5.1 (Schrödinger, USA). Potential hydrogen bond interactions with interatomic distances <3.0 Å were identified as constituent residues of the binding interface.

### Co-immunoprecipitation

Cells were transfected with *HDAC1-Flag* and *SATB1-Myc* overexpression plasmids either individually or in combination. Approximately 10 million cells were rinsed with PBS, scraped into 1 mL ice-cold lysis buffer (50 mM Tris-HCl pH 8.0, 137 mM NaCl, 1% Triton X-100, 1 mM EDTA, 10% glycerol, protease inhibitor cocktail), and incubated on ice for 30 min. After centrifugation at 20,000 × g for 15 min, cleared lysates were transferred to fresh tubes and incubated with the appropriate antibody overnight at 4°C with rotation. Immunoprecipitation was performed using 20 µL of anti-Myc magnetic beads (Bimake, #B26302) per sample. Bead–antigen complexes were washed three times with lysis buffer, resuspended in 50 µL of SDS loading buffer (Solarbio, #P1040), and heated at 95°C for 10 min.

Proteins were resolved by SDS-PAGE and electro-transferred to PVDF membranes (Bio-Rad, #1620177). Membranes were blocked for 1 h, then incubated overnight at 4°C with primary antibodies: anti-Myc (CST, #2276, 1:1 000), anti-FLAG (Sigma, #F7425, 1:2 000). Following three TBST washes, HRP-conjugated secondary antibodies—anti-mouse IgG (Abbkine, #A25112, 1:2 000) or anti-rabbit IgG (Proteintech, #SA00001-2, 1:3 000)—were applied. Immunoreactive bands were detected using an ECL substrate and captured with a Syngene G: BOX Chemi XX6 imaging system (Syngene, UK).

### Statistical analysis

Statistical analyses were performed using GraphPad Prism 10.1 (GraphPad Software, USA). All data are expressed as mean ± SD. *P* values were determined using an unpaired two-tailed Student’s t-test for two-group comparisons and one-way ANOVA for multiple-group comparisons. Significance indicators for each figure are detailed in the corresponding legends. A *P* value < 0.05 was considered statistically significant.

## Results

### *HDAC1* and *SATB1* respond to LPS-induced immune response in HD11 cells

**Fig. 1:**
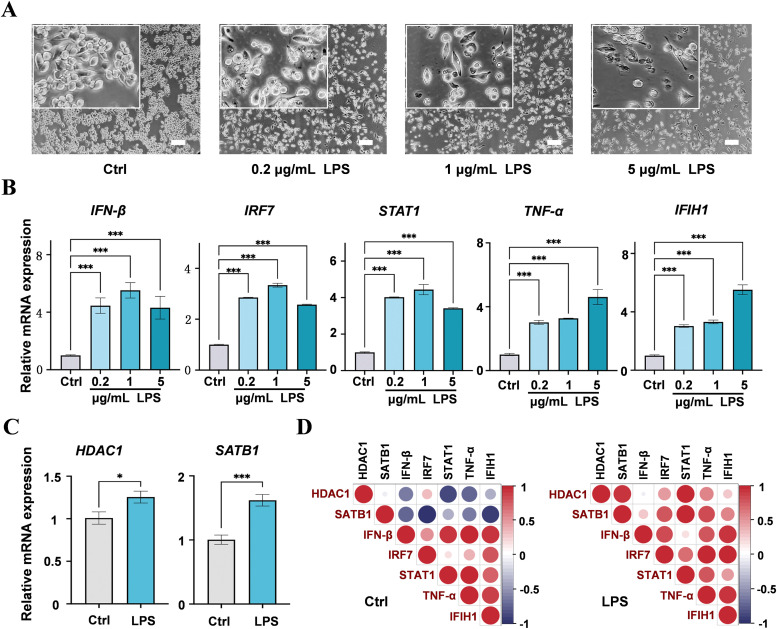
Immune responses of HD11 cells under LPS treatment. (A) HD11 cells were left untreated (Ctrl) or exposed to the indicated LPS. Enlarged images are shown in the top boxes. Scale bar, 100 μm. (B) RT-qPCR analysis of the expression levels of immune-related genes in HD11 cells under the indicated LPS. (C) RT-qPCR analysis of *HDAC1* and *SATB1* expression in HD11 cells treated with 1 μg/mL LPS. Data in (B, C) are presented as mean ± SD from 3 biological replicates per condition. *P < 0.05, ***P < 0.001. (D) Pearson correlation between the indicated genes in the control and 1 μg/mL LPS-treated groups. Red circles indicate positive correlation and blue circles indicate negative correlation. The size of the circle corresponds to the correlation coefficient.

Trace amounts of LPS are sufficient to elicit an immune response in chicken macrophages, whereas excessive concentrations become cytotoxic (Horii et al., 2025). To establish an immunocompetent model, HD11 cells were stimulated for 24 h with 0.2, 1 or 5 μg mL⁻¹ LPS and subsequently examined for morphological alterations and immune gene expression by RT-qPCR. Untreated control (Ctrl) cells grew as round, adherent monolayers; upon LPS exposure, the cells polarized and adopted irregular, spread-out shapes, accompanied by a progressive decline in viable cell numbers (Fig. 1A). At the transcriptional level, LPS treatment significantly upregulated key immune genes relative to controls. Concentrations of 0.2, 1, and 5 μg mL⁻¹ LPS strongly induced *IFN-β, IRF7, STAT1, TNF-α* and *IFIH1* (Fig. 1B; *P* < 0.001). In the 5 μg mL⁻¹ LPS group, the up-regulation of *TNF-α* and *IFIH1* was higher than that in the 1 μg mL⁻¹ group, whereas *IFN-β, IRF7*, and *STAT1* were lower (Fig. 1B). These data indicate that the LPS-induced immune response is not entirely dose-dependent. Integrating cellular viability and gene expression data, stimulation with 1 μg mL⁻¹ LPS for 24 h was selected as the optimal condition for constructing the chicken macrophage immune-response model. Under these conditions, *HDAC1* and *SATB1* transcripts were significantly upregulated (Fig. 1C; *P* < 0.05 and *P* < 0.001, respectively), implicating both factors in the HD11 response to LPS. Subsequently, the correlation in expression among *HDAC1, SATB1* and the immune effector genes was analyzed; strong positive correlations were observed in the LPS-treated group (Fig. 1D), suggesting that *HDAC1* and *SATB1* expression is closely linked to the transcriptional up-regulation of these immune effectors.

### Disruption of HDAC1 and SATB1 in HD11 cells through CRISPR/Cas9-mediated gene editing

**Fig. 2:**
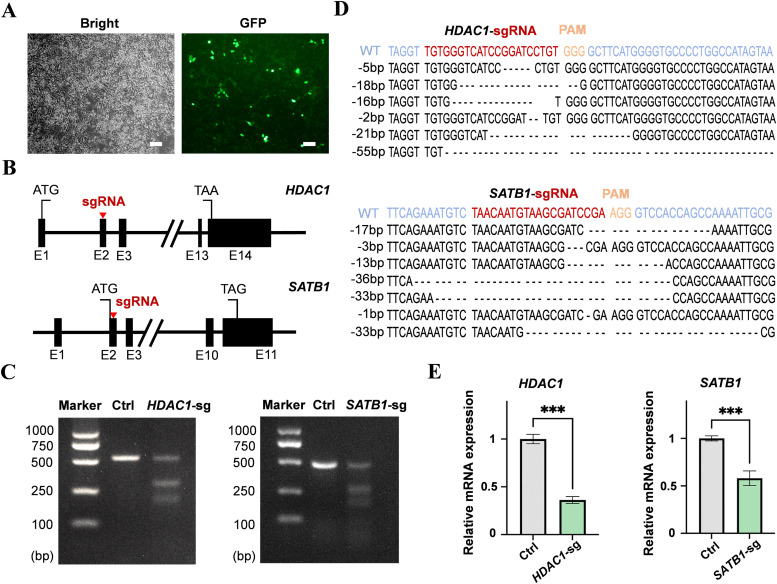
CRISPR/Cas9-mediated disruption of *HDAC1* and *SATB1* in HD11 cells. (A) Bright-field and green-fluorescence images of HD11 cells after transfection with the *eGFP* plasmid. Scale bar, 100 µm. (B) Schematic of the CRISPR/Cas9 target sites in *HDAC1* and *SATB1*. Red triangles indicate target locations; E, exon. (C) Representative T7E1 assay gels showing CRISPR/Cas9-induced indels in *HDAC1* and *SATB1*. PCR products were amplified from genomic DNA of control (Ctrl), *HDAC1*-edited (*HDAC1*-sg), or *SATB1*-edited (*SATB1*-sg) HD11 cells. (D) Representative sequence alignments of *HDAC1* or *SATB1* indels generated by the CRISPR/Cas9 system. Red letters, target sites; orange letters, PAM sequences. Dashed lines indicate deleted nucleotides. Number on the left with (-) indicate the size of the deletion. (E) RT-qPCR analysis of knockdown efficiency for *HDAC1* and *SATB1* in the indicated cells. Data are presented as mean ± SD from 3 biological replicates per condition. ***P < 0.001.

To determine the regulatory roles of *HDAC1* and *SATB1* in HD11 cells, their expression levels were genetically perturbed. First, an *eGFP* plasmid was transfected into HD11 cells via nano micelle-mediated transfection, resulting in stable expression of green fluorescent protein (Fig. 2A), confirming that HD11 cells are competent for transfection and subsequent genetic manipulation. Next, specific target sites for *HDAC1* and *SATB1* were designed (Fig. 2B) and the corresponding CRISPR/Cas9 systems were constructed. After transfecting HD11 cells with these systems, followed by drug selection and cell expansion, T7E1 assays demonstrated efficient mutagenesis (Fig. 2C), with estimated editing efficiencies of 67.9% and 52.9% for *HDAC1* and *SATB1*, respectively. Mutant sequences were subsequently identified (Fig. 2D). RT-qPCR revealed that the mRNA levels of *HDAC1* and *SATB1* were reduced by 63% and 42%, respectively (Fig. 2E; *P* < 0.001). Although effective antibodies against chicken HDAC1 or SATB1 were unavailable, mass spectrometry quantification confirmed that the corresponding protein levels were reduced after gene editing (Fig. S1; *P* < 0.05), collectively demonstrating disruption of the target genes.

### HDAC1 and SATB1 positively regulate LPS-induced immune response in HD11 cells

**Fig. 3:**
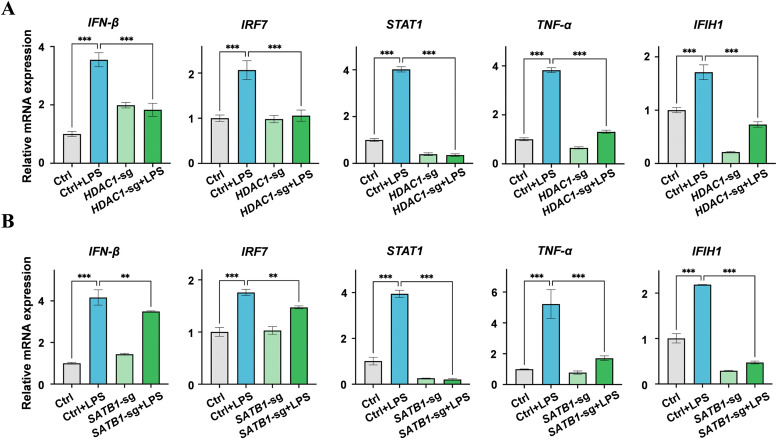
*HDAC1* and *SATB1* are required for the immune response in HD11 cells. (A) RT-qPCR analysis of immune-related gene expression in control and *HDAC1*-edited HD11 cells under the indicated LPS treatments. (B) RT-qPCR analysis of immune-related gene expression in control and *SATB1*-edited HD11 cells under the indicated LPS treatments. Data are presented as mean ± SD from 3 biological replicates per condition. **P < 0.01, ***P < 0.001.

To investigate the precise roles of *HDAC1* and *SATB1* in the immune response of HD11 cells, control cells and those with CRISPR/Cas9-mediated disruption of *HDAC1* or *SATB1* were stimulated with LPS, and the expression of immune-related genes was quantified. LPS treatment markedly up-regulated *IFN-β, IRF7, STAT1, TNF-α*, and *IFIH1* mRNA levels in control cells; in contrast, induction of these genes was significantly attenuated in *HDAC1*- or *SATB1*-depleted cells, with transcript levels substantially lower than those observed in LPS-treated controls (Fig. 3A, B; *P* < 0.01). To further comfirm the role of HDAC1 in the immune response, HD11 cells were treated with sodium butyrate (NaBt), a selective HDAC inhibitor. NaBt significantly suppressed the induction of immune-related genes after LPS challenge (Fig. S2; *P* < 0.001). Moreover, ectopic expression of *HDAC1* or *SATB1* largely restored the expression of immune genes that had been down-regulated by their respective depletion (Fig. S3, S4). Together, these data indicate that both HDAC1 and SATB1 act as positive regulators required for the immune response of HD11 cells.

### HDAC1 and SATB1 are highly conserved and act synergistically

**Fig. 4:**
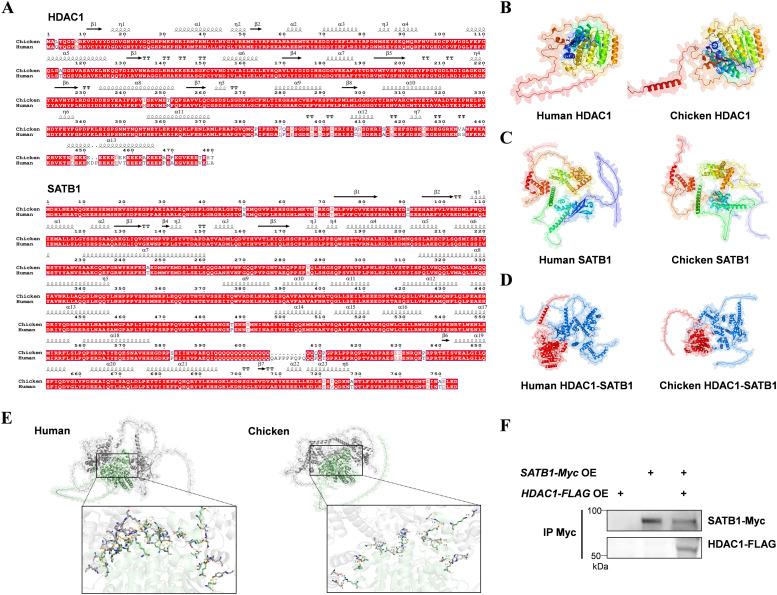
HDAC1 and SATB1 are highly conserved and act synergistically. (A) Pairwise alignment of amino-acid sequences: chicken HDAC1 versus human HDAC1, and chicken SATB1 versus human SATB1. Identical sequences are highlighted in red; predicted structural features are indicated above the alignment. (B) Predicted structures of human and chicken HDAC1. (C) Predicted structures of human and chicken SATB1. (D) ZDOCK-predicted structures of human and chicken HDAC1-SATB1 complexes. (E) AlphaFold3-predicted structures of human and chicken HDAC1-SATB1 complexes. Enlarged view highlighting the binding interface residues. (F) Immunoprecipitation followed by immunoblotting reveals that chicken HDAC1 interacts with chicken SATB1.

HDAC1 and SATB1 are generally regarded as evolutionarily conserved proteins (Lo Piccolo et al., 2023; Lozano et al., 2023; Zupkovitz et al., 2018). To assess their functional equivalence between human and chicken, we compared their amino-acid sequences and predicted three-dimensional structures. Pairwise alignments revealed 94% sequence identity for HDAC1 and 97% for SATB1 between the two species (Fig. 4A). Consistent with these findings, the in-silico-modelled structures of both proteins were highly similar (Fig. 4B, C). Therefore, it is speculated that chicken HDAC1 and SATB1 regulate immune responses in chicken HD11 macrophages in a manner comparable to that of their mammalian orthologs.

Because an interaction between mammalian HDAC1 and SATB1 has been reported (Kumar et al., 2006; Purbey et al., 2009; Yasui et al., 2002), we next assessed whether this interaction is conserved in chickens. Protein-protein docking was performed with ZDOCK to compare chicken HDAC1-SATB1 with human HDAC1-SATB1 complexes. Notably, the docking score for the chicken complex (1 497.2) was slightly higher than that for the human complex (1 376.6) (Fig. 4D). In addition, AlphaFold3 predictions yielded consistent results. The chicken HDAC1–SATB1 complex exhibited slightly higher confidence scores (ipTM: 0.33, pTM: 0.42) than the human HDAC1–SATB1 complex (ipTM: 0.25, pTM: 0.40) (Fig. 4E). Co-IP experiments performed on lysates from cells expressing both FLAG-HDAC1 and Myc-SATB1 validated the interaction between the two chicken proteins (Fig. 4F). Collectively, these findings indicate that chicken HDAC1 and SATB1 are structurally and functionally conserved and likely act in concert to regulate immune responses.

## Discussion

In this study, we unraveled, for the first time, the immune regulatory mechanisms mediated by HDAC1 and SATB1 in avian macrophages. Using an in vitro immune model based on the LPS-stimulated chicken macrophage cell line HD11, we observed two key phenomena: (1) LPS markedly up-regulated *HDAC1* and *SATB1* expression, and (2) CRISPR/Cas9-mediated disruption of either gene significantly attenuated the expression of key immune effector molecules (*IFN-β, IRF7, STAT1, TNF-α*, and *IFIH1*). These findings suggest that HDAC1 and SATB1 act as early-response factors during immune activation in avian macrophages and exert a potent positive regulatory influence on core avian immune pathways. Notably, the 1 µg mL⁻¹ LPS regimen used here imitates the sub-clinical, low-grade challenge ubiquitous on farms (Qin et al., 2023; Zhang et al., 2025), ensuring the findings translate directly to industry-relevant conditions.

HDAC1, the catalytic core of histone deacetylase complexes, is canonically linked to transcriptional silencing (Hayakawa and Nakayama, 2011; Laherty et al., 1997). During immune responses, HDAC1 exerts context-dependent activities that range from immunosuppression to immune potentiation, reflecting a complex regulatory landscape. For example, Bhat et al. (2024) demonstrated that inhibition of HDAC1 reprograms macrophage polarization toward an enhanced innate response against immunological stressors. De Sá Fernandes et al. (2024) reported that HDAC1-deficient dendritic cells acquire a hyperactive phenotype characterized by elevated immune capacity. While Rica et al. (2025) revealed that HDAC1 controls the generation and maintenance of effector-like CD8+ T cells, a process critical for limiting viral load during chronic infection, and Shao et al. (2020) showed that loss-of-function mutations in the Caenorhabditis elegans orthologue HDA-1 increase susceptibility to pathogenic bacteria and accelerate death, whereas transgenic overexpression of HDA-1 confers resistance and improves survival. Collectively, these reports underscore the concept that a single epigenetic regulator can preserve immune homeostasis through divergent molecular networks across vertebrate species. The role of HDAC1 in the avian immune response remains to be systematically characterized.

While our data demonstrate that HDAC1 and SATB1 are essential for the upregulation of key immune genes such as *IFN-β, IRF7, STAT1, TNF-α*, and *IFIH1* in LPS-stimulated chicken macrophages, the precise molecular mechanism—whether these genes are direct transcriptional targets of HDAC1/SATB1 or are regulated through secondary signaling cascades—remains to be fully elucidated. SATB1 has been characterized as a global chromatin organizer that orchestrates the spatio-temporal expression of gene clusters across large genomic distances (Zelenka and Spilianakis, 2020). Our cross-species sequence/structure comparisons reveal strong evolutionary conservation of HDAC1 and SATB1, and both in silico prediction and co-IP demonstrate an interaction between chicken HDAC1 and SATB1, suggesting that this regulatory module may be preserved in vertebrate immunity. Because SATB1 establishes chromatin loops, it may, through its unique nuclear-matrix-binding capacity, recruit HDAC1 to discrete loci—such as the promoters or enhancers of immune genes—thereby modulating local histone acetylation and fine-tuning transcriptional output in chicken macrophages challenged with LPS. In this context, immune genes such as *STAT1* and *IFN-β*—which form a feedback loop in key innate immunity—could be direct targets of an HDAC1-SATB1 regulatory module, given their rapid induction upon LPS challenge and the attenuated response in knockout cells. However, we cannot rule out the possibility that HDAC1 and SATB1 exert their effects indirectly, for instance by regulating upstream transcription factors or modulating signaling pathways, which subsequently activate immune effector genes. Future studies employing chromatin immunoprecipitation followed by sequencing (ChIP-seq) or CUT&Tag assays in chicken macrophages will be essential to map the genome-wide binding sites of HDAC1 and SATB1 and to determine whether they directly associate with the regulatory regions of the immune genes identified here.

The LPS-induced upregulation of immune genes did not exhibit a simple linear dose–response relationship, suggesting the presence of complex regulatory circuits that modulate the immune output. First, high-dose LPS may trigger feedback inhibitory pathways, such as the induction of suppressors of cytokine signaling or anti-inflammatory cytokines, which dampen the transcription of certain pro-inflammatory genes. Second, excessive LPS can provoke cellular stress responses, including oxidative stress and endoplasmic reticulum stress, which are known to interfere with transcriptional fidelity and mRNA stability of immune mediators. Third, differential activation thresholds among downstream signaling cascades may lead to preferential gene expression at particular LPS concentrations. Finally, receptor saturation or desensitization at high LPS levels could alter signal transduction kinetics and downstream gene-expression profiles. Although the present study did not experimentally dissect these possibilities, the observed non-linearity highlights the sophistication of avian macrophage responses and warrants further investigation into the feedback and stress-modulated networks that shape immune gene transcription.

Additional limitations of this study merit consideration. First, our findings are currently restricted to the HD11 cell line and await validation in primary chicken macrophages and in vivo tissues. In vitro models cannot adequately recapitulate the full complexity of the immune microenvironment, particularly intercellular interactions and hormonal regulation. Second, these findings remain to be validated against other avian pathogens, including avian influenza virus and Salmonella (Drauch et al., 2022; Hassan and Sharif, 2025). Third, while the AlphaFold prediction met the baseline confidence score threshold (>0.3), the relatively low confidence value suggests that the modeling may be incomplete. Notably, mammalian SATB1 recruits not only HDAC1 but also ISWI and ACF1 (Yasui et al., 2002); therefore, the prediction may lack additional cofactors or DNA templates required for accurate complex assembly. Consequently, experimental validation of the complete HDAC1–SATB1 multiprotein complex (e.g., co-IP followed by mass spectrometry) is required. Finally, the downstream effector molecules of this axis remain to be elucidated.

Notably, although HDAC1 and SATB1 have been implicated in mammalian immune regulation, their roles in avian immunity remained unexplored. Here we demonstrate that HDAC1 and SATB1 constitute an evolutionarily conserved functional module in chicken macrophages that boosts host defense against pathogens by up-regulating key immune mediators such as IFN-β and TNF-α. In silico analyses and co-IP further verify an interaction between chicken HDAC1 and SATB1, mirroring observations in mammals. Collectively, our findings extend the HDAC1-SATB1 regulatory axis to avian species, provide the first evidence for their synergistic enhancement of innate immunity in poultry, and improve a molecular framework for future strategies to improve disease resistance —advances that could ultimately support sustainable farming by reducing reliance on antibiotics.

## Conclusion

The current study established an LPS-stimulated immune model in chicken macrophage HD11 cells and generated stable HD11 lines with either *HDAC1* or *SATB1* constitutively knocked down. Functional analyses revealed that both HDAC1 and SATB1 act as pivotal regulators within the chicken innate immune network: upon LPS challenge, their expression was markedly up-regulated, and silencing either gene significantly attenuated the transcription of key effector molecules—including IFN-β, IRF7, STAT1, TNF-α and IFIH1—indicating that both genes positively modulate the host response to pathogen-associated molecular patterns. Protein complex prediction and co-IP further revealed that chicken HDAC1 and SATB1 interact, providing structural and physical insights into their cooperative transcriptional regulation. Collectively, these findings clarify the regulatory roles of HDAC1 and SATB1 in the chicken innate immune system, expand the theoretical framework of poultry immune regulation, and identify potential molecular targets for the genetic improvement of disease resistance in poultry.

## Data availability

The data and materials supporting this study are available from the corresponding author upon reasonable request.

## CRediT authorship contribution statement

**Bowen Niu:** Writing – original draft, Visualization, Validation, Methodology, Investigation, Data curation. **Junda Hu:** Writing – original draft, Visualization, Validation, Methodology, Investigation, Formal analysis, Data curation. **Zixuan Fan:** Writing – review & editing, Visualization, Validation, Software, Investigation, Formal analysis, Data curation. **Zihao Gao:** Writing – review & editing, Methodology, Investigation, Data curation. **Yuchen Jie:** Writing – review & editing, Methodology, Investigation, Conceptualization. **Xinyu Wu:** Writing – review & editing, Investigation. **Xingying Chen:** Writing – review & editing, Investigation. **Sirui Chen:** Writing – review & editing, Supervision, Resources, Funding acquisition. **Li-Wa Shao:** Writing – original draft, Validation, Supervision, Resources, Project administration, Methodology, Investigation, Funding acquisition, Conceptualization.

## Disclosures

The authors declare that they have no known competing financial interests or personal relationships that could have appeared to influence the work reported in this paper.

## Appendix Supplementary materials

Image, application 1

Image, application 2

Image, application 3

## Footnotes

Supplementary material associated with this article can be found, in the online version, at doi:10.1016/j.psj.2026.106607.
