# Supplementary material for: HDAC1 and SATB1 positively regulate immune responses in chicken macrophages
Source: Poult Sci. 2026 Feb 10;105(5):106607. doi: 10.1016/j.psj.2026.106607 (PMC12925555; doi:10.1016/j.psj.2026.106607)
Supplement: Supplementary file 1 [file mmc1.pdf]

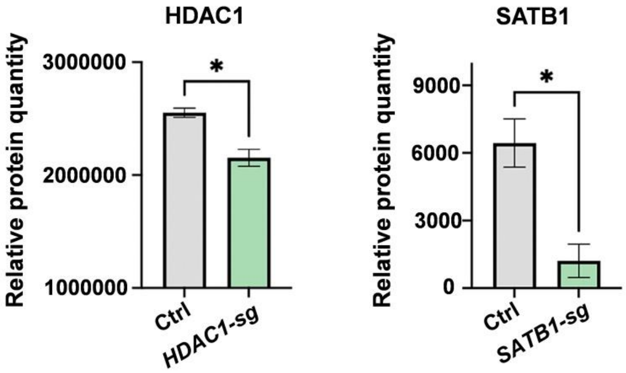

Fig. S1. CRISPR/Cas9-mediated gene editing downregulates HDAC1 and SATB1 protein levels. Quantification of HDAC1 and SATB1 in control (Ctrl) and gene-edited (sg) cells. Data are presented as mean ± SD from 2 biological replicates per condition. *$P < 0.05$.

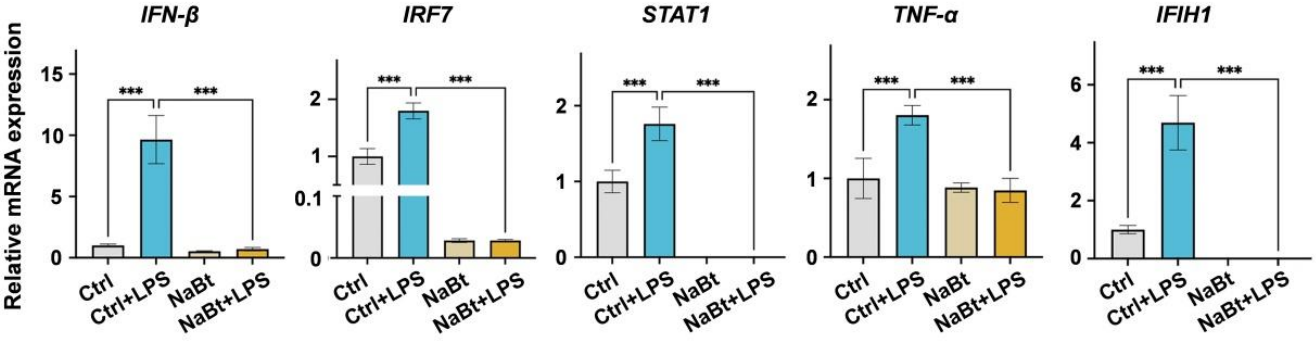

Fig. S2. Sodium butyrate inhibits LPS-induced immune responses in HD11 cells. RT-qPCR analysis of immune-related gene expression in control and NaBt-treated HD11 cells under LPS treatment. Data are presented as mean ± SD from 3 biological replicates per condition. ***$P < 0.001$.

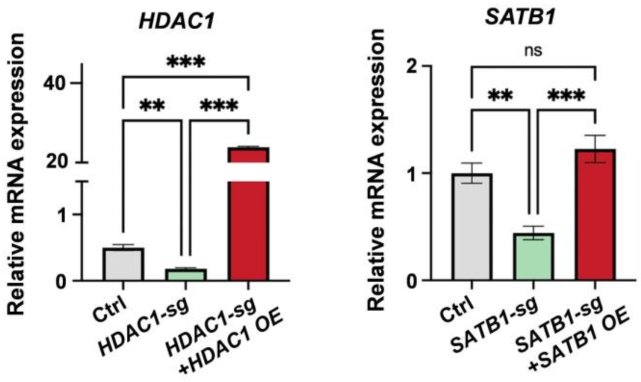

Fig. S3. Ectopic expression of *HDAC1* and *SATB1* rescues the down-regulation caused by gene editing. RT-qPCR analysis of *HDAC1* and *SATB1* mRNA levels in the indicated cells. OE, overexpression. Data are presented as mean ± SD from 3 biological replicates per condition. ***P* < 0.01, ****P* < 0.001; ns, not significant.

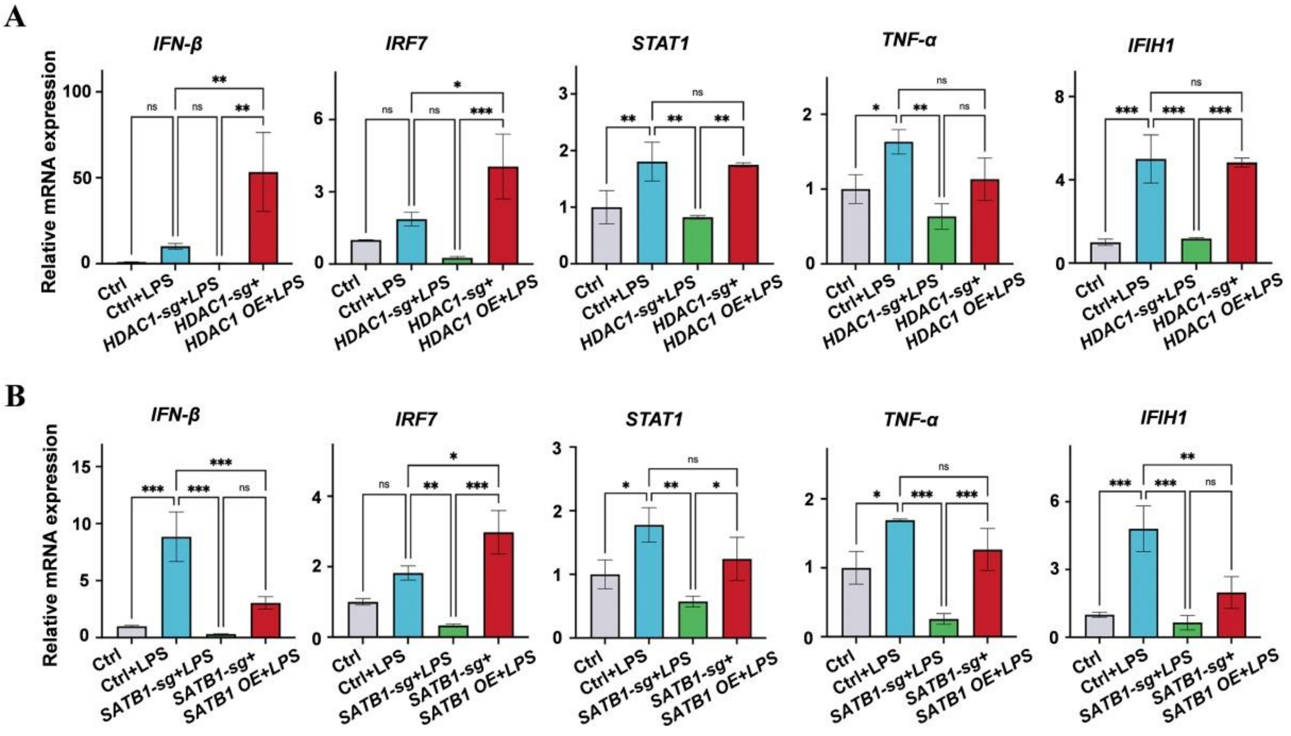

Fig. S4. Ectopic expression of *HDAC1* and *SATB1* restored the immune response in HD11 cells that had undergone the same gene editing. (A) RT-qPCR analyses of immune-related gene expression in HD11 cells that were either untreated controls, subjected to *HDAC1* editing, or subjected to *HDAC1* editing plus *HDAC1* overexpression, followed by LPS treatment. (B) RT-qPCR analyses of

immune-related gene expression in HD11 cells that were either untreated controls, subjected to *SATB1* editing, or subjected to *SATB1* editing plus *SATB1* overexpression, followed by LPS treatment. Data are presented as mean ± SD from 3 biological replicates per condition. *$P < 0.05$, **$P < 0.01$, ***$P < 0.001$, ns, not significant.
