# Supplementary material for: HDAC1 and SATB1 positively regulate immune responses in chicken macrophages
Source: Poult Sci. 2026 Feb 10;105(5):106607. doi: 10.1016/j.psj.2026.106607 (PMC12925555; doi:10.1016/j.psj.2026.106607)
Supplement: Supplementary file 3 [file mmc3.pdf]

Table S1. Primer sequences used for RT-qPCR

Gene	Primer Sequence (5'→3')
ACTB	F: GAGAAATTGTGCGTGACATCA<br>R: CCTGAACCTCTCATTGCCA
IFN-β	F: CTTGCCCACAACAAGACGTG<br>R: TGTTTTGGAGTGTGTGGGCT
IRF7	F: CAGGGCCACAGTTTCAGGAT<br>R: GGGATGGCACATTCCTCACT
STAT1	F: TCTGGAACGATGGCTGTA<br>R: GCTCCTTCTTGGTGTATGG
TNF-α	F: GGGACATCTCTCCAGGGGAA<br>R: CGAGCACTGCATTGTGTCT
IFIH1	F: GATTACCAGATGGAAGTTGC<br>R: GGTAATGTAAACAGCCACTC
HDAC1	F: GGTGGCTATACGATCCGCAA<br>R: GAGGGACTGATGTGCAGCTT
SATB1	F: CTGCCAGAAGCTGAACGAGA<br>R: TTGCAAACAGCGCTTGAGAC
